# 6^th ^International Symposium on Retroviral Nucleocapsid

**DOI:** 10.1186/1742-4690-5-21

**Published:** 2008-02-25

**Authors:** Ben Berkhout, Robert Gorelick, Michael F Summers, Yves Mély, Jean-Luc Darlix

**Affiliations:** 1 Laboratory of Experimental Virology, Department of Medical Microbiology, Center for Infection and Immunity Amsterdam (CINIMA) Academic Medical Center of the University of Amsterdam K3-110, Meibergdreef 15, 1105 AZ Amsterdam, The Netherlands; 2 AIDS Vaccine Program SAIC-Frederick, Inc. NCI-Frederick P.O. Box B Frederick, MD 21702-1201, USA; 3 Department of Chemistry and Biochemistry and Howard Hughes Medical Institute, University of Maryland Baltimore County, 1000 Hilltop Circle, Baltimore, MD 21250, USA; 4 Départment Pharmacologie et Physico-chimie, UMR 7175 CNRS, Institut Gilbert Laustriat, Université Louis Pasteur, 74 route du Rhin, 67401 Illkirch, France; 5 LaboRetro INSERM #758, Ecole Normale Supérieure de Lyon, IFR 128 Biosciences Lyon-Gerland, 69364 Lyon Cedex 07, France

## Abstract

Retroviruses and LTR-retrotransposons are widespread in all living organisms and, in some instances such as for HIV, can be a serious threat to the human health. The retroviral nucleocapsid is the inner structure of the virus where several hundred nucleocapsid protein (NC) molecules coat the dimeric, genomic RNA. During the past twenty years, NC was found to play multiple roles in the viral life cycle (Fig. 1), notably during the copying of the genomic RNA into the proviral DNA by viral reverse transcriptase and integrase, and is therefore considered to be a prime target for anti-HIV therapy. The 6^th ^NC symposium was held in the beautiful city of Amsterdam, the Netherlands, on the 20^th ^and 21^st ^of September 2007. All aspects of NC biology, from structure to function and to anti-HIV vaccination, were covered during this meeting.

## Overview

**Figure 1:**
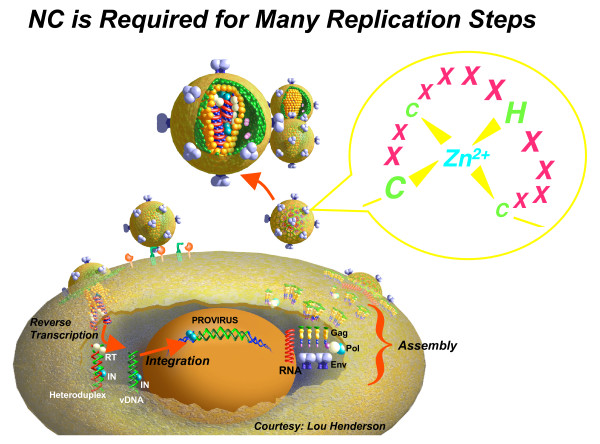
**NC protein is required for many replication steps**. Mature NC molecules coat the genomic RNA, in a dimeric form, in the viral particle and thus exert an essential function in virion structure. As illustrated in this cartoon – courtesy of Dr. Lou Henderson (NCI-Frederick, MD, USA) – NC protein chaperones conversion of the genomic RNA into the viral DNA by the RT enzyme. NC is also involved in the maintenance of the newly made viral DNA and in its integration into the host genome to form the provirus. In the course of virus assembly, the NC domain of Gag pilots genomic RNA selection which in turn acts as a scaffold for Gag oligomerization and assembly. As highlighted in the top circle, the CCHC zinc finger is central to all the NC-mediated functions. Thus, the retroviral zinc finger motif is viewed as a major target in anti-HIV drug development.

In 1998, Larry O. Arthur and Louis E. Henderson of NCI-Frederick decided that the field of nucleocapsid (NC) research was at a stage in which an NC symposium would be very useful. There was a general realization that NC was central to many processes in retrovirus replication and the highly conserved NC Zn^2+^-fingers were necessary for genome packaging and early infection processes (Fig. 1). Drs Arthur and Henderson hosted the first symposium at NCI-Frederick, MD, USA, (June 1998) and termed it International Retroviral Nucleocapsid Symposium (IRNCS) to be sure to include all the significant work being conducted on NC throughout the world. A series of meetings have been held since the inception of the initial symposium, namely the 2^nd ^IRNCS in September 1999 (J-L Darlix, ENS Lyon, France), 3^rd ^IRNCS in October 2001 (L. O. Arthur, Loews Annapolis, MD, USA), 4^th ^IRNCS in September 2003 (Y. Mély, Faculté de Pharmacie, Strasbourg, France), and 5^th ^IRNCS in September 2005 (L. Kleiman, McGill University, Montreal, Quebec, Canada). The 6^th ^IRNCS was held in Amsterdam to discuss the most recent advances on the functions of the NC protein in the synthesis, maintenance and integration of the proviral DNA, and in virus particle assembly. All these topics have been covered at the meeting to gain a better understanding of the multifunctional nature of NC (Fig. 1). Moreover, recent findings on other viral and cellular proteins playing a role in the viral life cycle that are either associated with NC, or resemble it in structure or function, such as the antiviral APOBEC3G protein, have been discussed at the conference.

## NC structure and its relationship to function

This first session on NC structure-function relationships discussed the basis of NC recognition of the genomic RNA and its potent nucleic acid chaperoning activities, discovered in the late 80's [1-6] (for reviews see [7-9]). Using single DNA molecules that are stretched and melted by force, M. Williams (Northeastern University, Boston, MA, USA) and collaborators examined the influence of HIV and HTLV-1 NC on double stranded DNA destabilization and reannealing. It was found that HIV NC behaved as a pure chaperone while HTLV-1 NC was more akin a single stranded DNA binding protein [10-12] in agreement with the recent findings of K. Musier-Forsyth (OSU, Columbus, OH, USA; see below). Based on standard assays to monitor the NC nucleic acid binding, aggregation and annealing activities, K. Musier-Forsyth and collaborators showed that the N-terminal 35 residues of HIV-1 NC were necessary and sufficient for chaperone function *in vitro*. Interestingly, the HIV-1 Gag polyprotein was also shown to be a nucleic acid chaperone protein, a property that is likely to facilitate genome dimerization and tRNA primer annealing during virion assembly. Results on different retroviral NC proteins showed that they exhibit significant differences in their overall chaperone activity, decreasing in the order HIV-1 ~RSV > MLV >> HTLV-1. Both K Musier-Forsyth and M Williams found that HTLV-1 NC's poor chaperone activity was caused by its acidic C-terminal domain. Using fluorescence-based techniques and the HIV-1 TAR stem-loop (SL) structure, Y Mély and his colleagues (Faculté de Pharmacie, Strasbourg, France) showed that the HIV-1 NC structural determinants, formed by the hydrophobic plateau at the top of the two zinc fingers and the flanking basic residues, are essential for its chaperoning function [13-15]. Similar structural determinants were also found to be essential for the chaperoning function of MoMLV NC that contains a unique zinc finger that is also flanked by basic residues [16].

## Retroviral RNA structures and functions, and RNA-NC interactions

**Figure 2:**
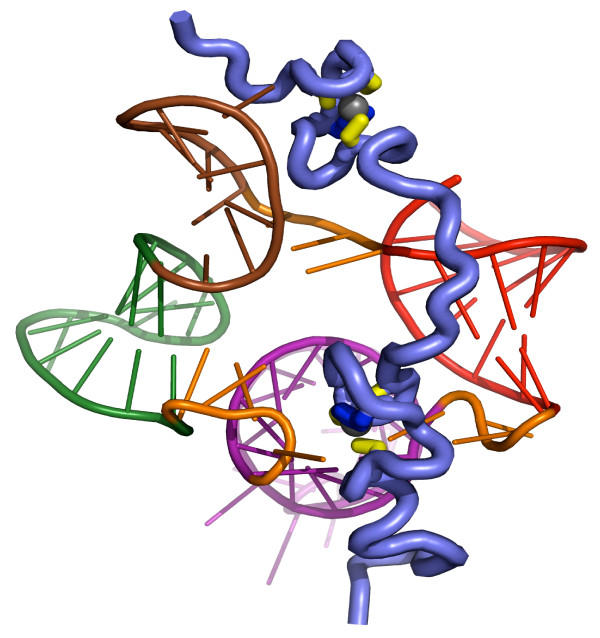
**Structure of RSV NCp12 bound to the muPsi packaging signal**. Structure of the Rous sarcoma virus nucleocapsid protein NCp12 bound to the cognate muPsi RNA packaging signal. The backbone of NC is shown as a ribbon (slate) and the zinc, cysteine and histidine groups are colored silver, yellow and blue, respectively. Coloring of secondary structural elements of the muPsi RNA are: SLA, purple; SLB, green; SLC, brown; O3, red; linker segments, orange (20).

Several retroviral RNA structures, such as the 5' untranslated region (5'UTR) or leader, are the subject of intense interest because they are involved in the early and late phases of virus replication via multiple RNA-protein interactions, notably with NC. During retrovirus assembly NC, as the C-terminal domain of Gag, plays an essential role in specifically recruiting two copies of the full length genomic RNA and causing its dimerization. Deciphering the 3D structure of the Psi RNA packaging signal in the the 5' UTR is key to our understanding of NC-genomic RNA interactions. To that end the group of M. Summers (UMBC, Baltimore, MD, USA) is investing much effort to establish the first high resolution model of the 100 nucleotide core encapsidation signal (Ψ^CES^) of MoMuLV, which comprises stem loops (SL) B-D. Through a combination of nucleotide specific, segmental labeling, and sub-fragment analysis, they have obtained high quality and high resolution NMR (nuclear magnetic resonance) spectra indicative of dimer formation. In particular A289 and A293, which are part of the hairpin loop in SLB, give rise to signature peaks and nuclear Overhauser effect (NOE) patterns upon RNA dimer formation. Residues A330 and A364, which are part of SLC and SLD, respectively, give rise to downfield peaks diagnostic of kissing interactions between SLC and SLD. Additionally, using a novel transverse relaxation-optimized spectroscopy (TROSY) based heteronuclear single quantum correlation (HSQC) pulse sequence, residual dipolar couplings have been measured for isolated B-duplexes and this method is currently being applied to the 198-nucleotide dimeric (Ψ^CES^) site. This work will lead to the first high resolution model of the dimeric Ψ^CES ^site. This group has also analysed interactions between the NC proteins and RNA packaging signals of other retroviruses, including MoMLV and Rous Sarcoma Virus (RSV). It was found that the native MoMLV Ψ allowed an average of 15 NCs to bind while mutant-Ψ, which can not form dimer, allowed only an average of two NC molecules to bind. These results show that exposure of NC binding sites by Ψ dimerization occurs in the entire Ψ-site, by which NC recognizes, selectively dimerizes, and packages its genomic RNA into the virion [17-19]. RSV is unusual in that its genome can be efficiently packaged by a relatively short, 82-nucleotide segment of the 5'-UTR called muPsi. Upon NC binding, muPsi adopts a stable secondary structure that consists of three stem loops (SL-A, SL-B and SL-C) and an 8-base pair stem (O3), (see Figure 2) [20].

In the forefront of developing new technologies for high-throughput and accurate RNA secondary structure analysis, K Weeks (UNC Chapel Hill, NC, USA) reported the first generation of these technologies which involves Selective 2'-Hydroxyl Acylation analyzed by Primer Extension (called SHAPE) [21-24]. Because SHAPE makes it possible to obtain RNA secondary and tertiary structure information for hundreds or thousands of nucleotides over a few days or weeks, it is now becoming possible to tackle several ambitious problems involving the role of RNA structure in retroviral biology. Because of its completeness, high-throughput SHAPE analysis provides information sufficient to discriminate between otherwise contradictory models for HIV-1 genome structure. SHAPE analysis, performed *in virio*, allowed a series of previously unrecognized specific NC binding sites to be identified. *In virio *analysis also facilitated detailed characterizations of both the specific RNA binding and the contrasting duplex destabilizing activity of NC protein. Further application of high-throughput SHAPE has significant potential to help establish the underlying connections between RNA structure and transcriptional and translational regulation and with other RNA-based processes in retroviral infectivity.

K. Purzycka and colleagues (Polish Academy of Sciences, Poznan, Poland) pursued their structural and dynamics analyses of the HIV-2 5' UTR RNA. They presented a new structure model for the DIS (dimer initiation site) of HIV-2 based on the high-throughput prediction of 3D RNA structures at low resolution [25,26] and molecular dynamic simulations [27].

Using functional assays A Das, M. Vrolijk and colleagues (B Berkhout, Amsterdam, the Netherlands) reinvestigated the role of the highly conserved TAR SL structure in HIV-1 replication [28]. They concluded that TAR has no essential function in HIV-1 biology other than to accommodate Tat-mediated activation of transcription [29]. But non-basepaired nucleotides at the 5' end of the 5' UTR can have adverse effects on its structure and functions in RNA dimerization and packaging mediated by the packaging signal (Psi or Ψ). They concluded that HIV-1 requires a stable SL structure at the start of the viral transcripts to avoid misfolding of the leader RNA so that it can fulfill all its functions.

A. Lever and colleagues (University of Cambridge, UK) have long since been interested in the mechanism of HIV-1 genomic RNA selection and packaging during Gag assembly, that relies on the 5' Ψ packaging signal formed of SL structures [30]. The tip of SL1 is a dimer linkage site that has a purine-rich bulge proximal to it. This and a proximal bulge in SL3 appear to be metastable, probably facilitating RNA unwinding when Gag protein binds. They have identified another purine rich bulge in the SL1 stem loop which binds to the regulatory Rev protein. Disrupting Rev binding impairs virus replication. Some of the variant models in the structure of the Ψ RNA reflect the fact that the RNA undergoes significant conformational changes during its trafficking through the cell to the viral particle [31].

## Role of NC in reverse transcription and recombination

NC is an obligatory constituent of the viral replication machinery whereby the genomic RNA is converted into the full-length double-stranded viral DNA by RT. This appears to be mediated by tight interactions between NC molecules, the genomic RNA that is in a dimeric form and RT. In a long standing effort to understand the role of NC in viral DNA synthesis, R Gorelick et al. (NCI-Frederick, MD, USA) [4] discussed their investigation of two HIV NC zinc-finger mutants, H23C and H44C, which exhibit severe replication defects [32]. Analyses of early infection revealed that these mutations cause major defects in integration, but not reverse transcription: curiously, the rate of initiation and partial completion of reverse transcription was faster than wild-type [33]. Examination of virus particles prior to infection revealed that both mutant virions contain significantly more viral DNA than wild-type particles. Thus these NC mutations cause premature reverse transcription, and this may provide a partial explanation for their replication defects.

J. Mak and colleagues (Macfarlane Burnet Centre, Victoria, Australia) investigated the rate of HIV-1 recombination in cell cultures by monitoring sequence-tag redistribution in the *gag *and *pol *genes. They found that rates of recombination were high, corresponding to about 6–7 per cycle in T cells and up to 12–14 in monocyte-derived macrophages.

In addition to the genomic RNA, HIV-1 virions can package a substantial amount of spliced viral RNAs, but this seems to be independent of the NC zinc fingers, as reported by M. Mougel et al. (CNRS, Montpellier, France). Reverse transcription of these spliced RNAs takes place as efficiently as that of the genomic RNA, but poorly responds to the chain terminator antiviral drug AZT. Thus, AZT treatment might well increase the representativeness of spliced HIV-1 DNAs [34,35].

All viral RNA, the full length and the spliced forms, contain the TAR sequence at the 5' and 3' ends. NC can induce TAR dimerization as reported by E. Andersen et al (University of Aarhus, Denmark) [36]. According to these authors, TAR dimerization is important for the obligatory first strand transfer during cDNA synthesis and this TAR dimer has a parallel orientation depending, at least in part, on the dimerization initiation site (DIS). Completion of viral DNA synthesis necessitates the second strand transfer which takes place at the level of the PBS sequence. N Morellet et al (CNRS Paris, France) investigated this strand transfer in HIV-1 by means of NMR and fluorescence studies. They found that NC, notably the zinc fingers, chaperones this reaction by modifying the conformation of the loops of both PBS (+) and (-) sequences, promoting the formation of a kissing complex and the subsequent annealing of the PBS (+) and (-) sequences.

## Gag assembly and the role of NC

The genomic RNA codes for the Gag and Gag-Pol polyprotein precursors which are synthesized by the host translation machinery. Several characteristics of the HIV-1 5' UTR, such as the 5' terminal TAR hairpin, its length and overall secondary structure, are likely to interfere with ribosomal scanning and suggest that translation is initiated by internal entry of ribosomes (IRES) [37,38]. To gain further insight in the mechanism of translation initiation on the HIV-1 5'UTR, T. Abbink, K. Arts and B. Berkhout (Amsterdam, the Netherlands) introduced upstream AUGs (uAUG) at different positions in the 5'UTR and determined the effect on the expression of a downstream reporter gene under control of the gag AUG. If ribosomal scanning initiates upstream of the uAUG, translation will start on the uAUG, thereby inhibiting the expression of the downstream reporter gene. This allowed determination of the window of ribosomal scanning on the HIV-1 5' UTR. Their studies show that the inserted uAUG inhibited reporter gene expression at every position in the 5'UTR, thus indicating that the entire 5' UTR is scanned. In addition, they show that reinitiation of translation on the HIV-1 5'UTR is quite efficient if the upstream open reading frame is short (less than 20 bases). This finding is in agreement with previous findings [39] but different from others showing that HIV Gag translation operates by an IRES mechanism [40].

HIV-1 Gag polyprotein contains all of the molecular determinants required for its intracellular trafficking, and its assembly and budding in the form of virus-like particles (VLPs). The role of NC in Gag assembly has been studied for almost twenty years [1-5]. A. Rein and his colleagues (NCI-Frederick, MD, USA) have characterized the assembly properties of chimeric proteins in which the NC (and p1 and p6) domain of HIV-1 Gag is replaced by dimerizing or trimerizing leucine- or isoleucine-zipper motifs, respectively. These proteins assemble in mammalian cells into virus-like particles (VLPs) with morphology nearly, but not quite, identical to those formed by wild-type Gag. These VLPs contain at least 10-fold less RNA than wild-type VLPs. Their buoyant density is somewhat lower than that of wild-type VLPs; this difference is consistent with the hypothesis that the packing of the chimeric proteins in these VLPs is the same as that of wild-type Gag. The chimeric proteins co-assemble efficiently with wild-type Gag; thus the function of RNA in normal VLP assembly is not to act as a "string" upon which Gag protein molecules are aligned. The chimeric proteins were expressed in bacteria. They did not assemble spontaneously, but could be induced to assemble into VLPs by addition of either RNA or inositol hexakisphosphate.

D. Muriaux, J.L. Darlix and collaborators (INSERM Lyon, France) have been studying the trafficking and the assembly of HIV-1 in human cells. Viral and cellular factors are involved in these processes, in particular the NC domain of Gag, the genomic RNA, and cellular proteins of the endocytic pathways, and membrane microdomains [41]. They found that HIV-1 can bud into intracellular vesicles in addition to the plasma membrane of T cells [42]. The NC zinc fingers of HIV-1 Gag were found to be critical determinants of Gag assembly and localisation in endosomes [43]. Furthermore, they reported the involvement of a tetraspanin web in T-lymphoblastic cells that serves as a platform for HIV-1 assembly (Grigorov B., V. Attuil-Audenis, F. Perugi, M. Nedelec, S. Watson, J.-L. Darlix, C. Pique, H. Conjeaud & D. Muriaux: IMPLICATIONS OF TETRASPANINS IN HIV-1 FORMATION in infected T-lymphoblastic cells., submitted).

NC protein can exist in different forms in newly made mature HIV-1 virions, namely NCp15 (NC-SP2-p6), NCp9 (NCp7-SP2) and fully processed NCp7. J.A. Thomas (NCI-Frederick, MD, USA) presented work of collaborators where they investigated the requirement for proteolytic maturation of NC by mutating the protease cleavage sites necessary for the production of mature NCp9 (NCp7-SP2) or NCp7. Interestingly, viruses tailored to make either NCp9 + p6 or NCp7 + SP2 + p6 were fully infectious and could replicate in H9 cells. In contrast, viruses that made either NCp15 or NCp7 + p1-p6 were replication defective. Because the replication block was principally manifested by a severe reduction in integration, it is likely that NCp9 has a role in the integration process [44] and has also been shown to facilitate in vitro concerted integration even better than NCp7 [45].

## Cellular and viral proteins associated with NC

Human APOBEC3G (hA3G) has been identified as an anti-HIV-1 host factor acting by deaminating the newly made cDNA. S. Cen and collaborators (Lady Davis Inst., Montreal, Canada) reported that hA3G inhibits HIV-1 reverse transcription independently of its editing activity. A reduction of 55% in early viral DNA synthesis by hA3G is correlated with a similar decrease in the tRNA^Lys3 ^priming, which requires a hA3G/NCp7 interaction. A greater reduction of ~95% in late DNA synthesis results from the hA3G-induced inhibition of DNA strand transfer, which is correlated with its ability to prevent RNaseH degradation of the template RNA [46-48].

Y. Iwatani et al (NIH, Bethesda, MD, USA) have independently investigated possible effects of hA3G on RT and NC function *in vitro*. NC-mediated annealing and RNase H cleavage were not affected, but A3G inhibited all RT-catalyzed elongation reactions, independent of hA3G's catalytic activity. These data taken together with complementary biophysical analyses led to the conclusion that deaminase-independent inhibition of reverse transcription is determined by critical differences in the nucleic acid binding properties of NC, A3G, and RT [49,51].

In an attempt to understand the possible interactions between HIV-1 NC and hA3G, P Henry et al (NCI, Frederick, MD, USA) expressed hA3G in insect cells and purified it using metal affinity, ion exchange and size exclusion chromatography. Immunoprecipitation of purified NC with purified A3G bound to magnetic beads in the presence and absence of RNA revealed that A3G pulls down NC more efficiently in the presence of RNA, with decreasing efficiency as excess RNA was added to dilute simultaneous binding. The effect of NC on A3G enzymatic activity was examined by adding purified NC to their scintillation proximity assay mixture at a ratio of 1 NC molecule per 2, 4 or 8 nucleotides of substrate DNA, either before or with the addition of A3G [50]. In all cases, NC was unable to reduce cytidine deamination of the substrate DNA. These data indicate that interactions between NC and A3G are indirect and mediated by an RNA bridge. In addition, the results suggest that concentrations of NC that are likely to be present in the reverse transcription complex do not interfere with A3G's cytidine deaminase activity.

J.C. Paillart (CNRS, Strasbourg, France) reported data on a putative new role of the viral factor Vif. Vif is a small basic protein essential to viral fitness and pathogenicity. Marquet, Paillart and coworkers recently showed that Vif preferentially binds to the 5' UTR of the HIV-1 genomic RNA [52] and to corresponding DNA sequences [53]. Now they report that Vif stimulates the early steps of reverse transcription (tRNA^Lys3 ^annealing, strong-stop DNA synthesis, first strand transfer) and the formation of loose RNA dimers, suggesting that Vif is an RNA chaperone [54] However, Vif is able to inhibit NC-induced reactions such as hybridization of tRNA^Lys3 ^and formation of tight RNA dimers while it collaborates with NC to increase RT processivity. Thus, Vif might negatively regulate NC-assisted maturation of the RNA dimer and early steps of reverse transcription during assembly, but these inhibitory effects would be relieved after viral budding, due to the preferential exclusion of Vif from virions.

## Antivirals and NC vaccines

In this session new anti-viral drugs targeting HIV-1 protease and NC were described. To combat the widespread development of HIV-1 protease resistance in AIDS patients, novel protease inhibitors (PI) with high potency against the known PI-resistant mutants have been developed. M. Nijhuis and collaborators (University of Utrecht, the Netherlands) investigated whether HIV-1 could yet again find a way to become less susceptible to these novel drugs [55]. *Ex vivo *selection experiments have shown that HIV-1 indeed can use an alternative mechanism by the selection of mutations in the NC/SP2 cleavage site instead of the viral protease. These changes cause resistance to all protease inhibitors by enhancing the processing efficiency of the altered substrate by wild type protease. Modulation of the cleavage efficiency of this site impacts both PI susceptibility and viral replication capacity. Further studies are required to determine to what extent NC/P2 cleavage site mutations may explain virological failure during PI therapy.

**Figure 3:**
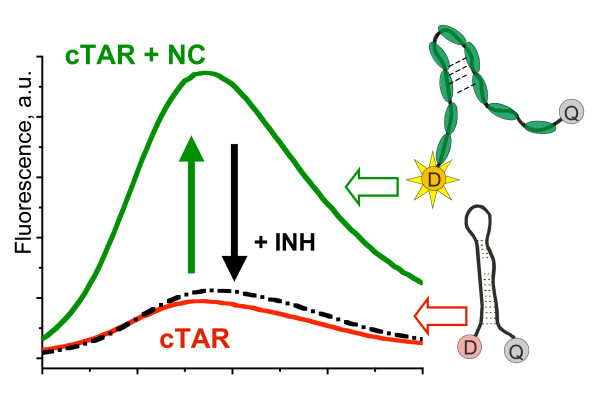
**Principle of the NC inhibitor screening assay**. This assay is based on the use of a HIV-1 TAR DNA (cTAR) sequence labeled at its 5' and 3' ends, by a fluorophore (D) and a quencher (Q), respectively. In the absence of NC, Q is close to D, leading to a small residual fluorescence of D. Due to its nucleic acid destabilizing properties, NC melts the cTAR structure up to the middle of the stem, leading Q to move away from D, thus causing a large fluorescence increase. A positive hit is detected through the restoration of the initial low fluorescence level.

Y. Mély (ULP, Strasbourg, France) in collaboration with J.L. Darlix (INSERM Lyon, France) and M. Gottikh (Moscow, Russia) developed a screening assay based on the ability of HIV-1 NCp7 to destabilize SL structures such as TAR (Fig. 3). Several lead molecules were identified that inhibit NC destabilization activity in the low μM range. These molecules bind to the NC zinc fingers but do not eject zinc. Small Trp-rich hexapeptides selected from phage display libraries can also inhibit the NCp7 destabilizing activity in the low μM range through competition with its target sequences [56-58]. Finally, peptide and oligonucleotide-based compounds targeting NCp7 with low nM activities have been found and they are currently being characterized *in vitro *and in cell culture assays.

P. Mahy from H-PHAR (Gosselies, Belgium) reported new results on HPH116, which is a zinc finger ejector targeting NCp7 protein. HPH116 is a modified synthetic form of azodicarbonamide produced under good manufacturing practices (GMP) conditions developed by H-PHAR. In a TZM-bl cell infection model, HPH116-treated viral particles were found to have lost their infectivity, with up to 100% inhibition at 1 μM HPH116. Viral inhibition was enhanced when the viral particles were pre-incubated with HPH116 at 0.25 μM and 0.1 μM Tenofovir (vs 5 μM Tenofovir alone), 0.3 μM AZT (vs 30 μM AZT alone), or with 0.125 μM T20. Thus, when HPH116 is combined with other known antiretroviral drugs, it can induce a dramatic increase in virus inhibition.

S-acyl-2-mercaptobenzamide thioester lead compounds [59] were investigated as topical microbicides by Miller-Jenkins et al. Initial studies with C. Cheng-Mayer (Aaron Diamond AIDS Research Center, New York, USA) have shown that a lead compound protects macaques from SHIV infection in a dual-infection model of vaginal transmission. Three sites in NCp7 were found to be important for reactivity and specificity: position x+1 (where x is Cys36), x+2, and x+9 [60,61]. Furthermore, a secondary intramolecular S to N acyl transfer, which occurs after the primary intermolecular acyl transfer from the thioester to Cys36, was demonstrated. This secondary transfer is irreversible and leads to loss of NCp7 structure and, most probably, its function.

An update on work involving covalent modification of free S-H groups on internal viral proteins by electrophilic reagents to generate whole inactivated retroviral virions with functional envelope glycoproteins was presented by J. Lifson (NCI-Frederick, MD, USA). Studies with 4-vinyl pyridine showed that covalent modification of NC, without appreciable modification of other virion proteins is sufficient for inactivation. Inactivated viruses are being used for electron tomographic ultrastructural studies of whole virions and envelope glycoprotein spikes [62]. In additions tomographic studies were presented showing the interaction of Env with receptors on T cells [63]. *In vitro*, inactivated virions effectively primed both HIV-1 specific responses by MHC-I restricted responses by naïve CD8+ T cells and MHC-II restricted responses by naïve CD4+ T cells. Progress on the use of such particles as a vaccine immunogen was also presented.

## Authors' contributions

BB organized the symposium. RG and JLD were co-organizers. MFS and YM provided figures 2 and 3, respectively. JLD wrote the report. BB, RG, MFS and YM corrected the report.
